# Faculty Time Allocation at Historically Black Universities and Its Relationship to Institutional Expectations

**DOI:** 10.3389/fpsyg.2021.734426

**Published:** 2021-10-13

**Authors:** Martha Escobar, Zebulon Kade Bell, Mohammed Qazi, Christian O. Kotoye, Francisco Arcediano

**Affiliations:** ^1^Department of Psychology, Oakland University, Rochester, MI, United States; ^2^Department of Psychology, Louisiana State University Alexandria, Alexandria, LA, United States; ^3^Department of Mathematics, Tuskegee University, Tuskegee, AL, United States

**Keywords:** effort distribution, expectations for tenure, faculty time allocation, HBCU faculty, tenure at HBCUs

## Abstract

University faculty divide their time into their main academic responsibilities, typically identified as teaching, research, service, and, at institutions with strong ties to their surrounding community, outreach. Most studies of time allocation have focused on faculty at Primarily White Institutions. The present study investigated how faculty at five Historically Black Universities (HBUs) allocate their time to their academic responsibilities. Data were analyzed based on their tenure status, gender, and representation in science, technology, engineering, and mathematics. Faculty estimated the percentage of time they currently allocate (*current*), the time they would ideally allocate (*ideal*), and the time they estimate their institution expects them to allocate (*expected*) to each academic responsibility. Across all demographics, there were discrepancies between current and ideal time allocation to research and teaching and, in some demographics, outreach. The greatest discrepancy between current and expected time allocation was observed in time allocated to research, with women and untenured faculty also showing a discrepancy in time allocated to teaching, and underrepresented faculty showing no discrepancies between current and expected time allocation. Women, untenured, and underrepresented faculty reported that their time allocation patterns were guided by external factors rather than personal preferences. The surveyed faculty also stated that the patterns of effort distribution expected to obtain tenure were not necessarily guided by the faculty handbooks at their institution. Although this study is limited by its relatively small sample size, it provides an insight into how faculty at HBUs divide their time and the reasons for them to do so.

## Introduction

College and University faculty divide their time among three different academic responsibilities (Easterly and Pemberton, 2008). The first of these roles is *teaching*, which includes sharing of content knowledge with students, developing course materials, designing curriculum, and advising and mentoring students. A second academic responsibility is *research*, or the advancement of knowledge in their area of expertise, which involves investigation, data analysis, and dissemination of disciplinary knowledge. Finally, *service*, or community-centered activities, includes participation in committees that assist with the effective functioning of their academic unit or institution and professional associations. In addition to these three traditional academic responsibilities, some institutions of higher education also develop close ties to their surrounding communities and, as a result, a fourth, *outreach* function has emerged, with the goal of extending educational and research programs to the community. In this paper, we provide a brief review of the literature on faculty time allocation, with the goal of discussing effort distribution among faculty in science, technology, engineering, and mathematics (STEM) at five Historically Black Universities (HBUs) in the southeastern United States.

Although the primary purpose of institutions of higher education is to promote knowledge through their teaching mission, failures in the educational role of these institutions came under question (e.g., Wingspread Group on Higher Education, 1993). Between 1972 and 1992, there was a significant change in professorial activities, with most faculty’s efforts being focused on research and research productivity (Milem et al., 2000). The shift to a focus on research is at least partly determined by institutional pressure to secure external funding (grants and contracts; Anderson and Slade, 2016). The concerns about institutions of higher education failing to serve students’ educational needs led to an increased interest in pedagogy and teaching effectiveness (e.g., setting student learning goals for each course), which forced faculty (especially at smaller institutions) to change how much time and effort they allocate to the activities that make up their professorial careers. For example, increased emphasis on out-of-classroom mentoring increases the time allocated to teaching activities (e.g., Bok, 1992). The redistribution of professorial responsibilities became a dividing factor among so-called Research 1 (R1) and Research 2 (R2) institutions (Carnegie Classification of Institutions of Higher Education, 2018), which are characterized by offering graduate programs and very high (R1) and high (R2) research activity, and smaller institutions which do not reach the research and funding amounts to place them into either category. R1 and R2 institutions tend to prioritize research above any other activity, whereas other institutions expect more effort to be allocated to teaching, service, and/or outreach. The framework created by R1 and R2 institutions has created institutions of higher education that strive for homogenization, resulting in an “institutional drift.” That is, smaller institutions try to emulate this emphasis on research activities as they “strive to gain greater status and prestige by attempting to resemble more closely those institutions that have already established a ‘legitimate’ high ranking position in the institutional hierarchy” (Milem et al., 2000, p. 456). However, grants tend to go to larger institutions with state-of-the-art facilities, reducing the available resources for smaller institutions and new faculty (Murray et al., 2016).

College and University faculty are unique workers because their primary commitment is to their field of expertise rather than the institution itself; however, the expectations of each institution can strongly influence how faculty allocate their time to each of their academic responsibilities (Anderson and Slade, 2016). These institutional factors interact with individual characteristics, such as gender, tenure status, academic field, and personal preferences (Link et al., 2008). Untenured faculty are constantly pressured to navigate the expectations for attaining tenure and promotion, which in most post-baccalaureate granting institutions include evidence of successful grant proposal writing (Fairweather, 2002; Easterly and Pemberton, 2008). Link et al. (2008) observed that tenured, senior, faculty at research institutions allocate their time based primarily on personal preferences, gradually decreasing their emphasis on research and increasing their emphasis on teaching (associate professors) or service (full professors) as the pressure for tenure and promotion decreases (but see Betsey, 2007 and discussion below).

There are also well-documented gender differences in time allocation. For example, based on data from the National Study of Postsecondary Faculty of the National Center for Education Statistics, faculty report working 50–60h/week, with female assistant professors working less time than male assistant professors (Jacobs and Winslow, 2004). Among this work time, females allocate less time than males to research, and more time than males to teaching and service, which may reflect personal preferences (Winslow, 2010) or a feeling of obligation to take on these responsibilities (Bellas and Toutkoushian, 1999; Link et al., 2008; Misra et al., 2012; Dahm et al., 2015; French et al., 2020). Female faculty are also more likely to have a working spouse/partner than male faculty, and they must divide their limited time among work and family responsibilities (French et al., 2020). Indeed, faculty (predominantly female) who provide care for children or other family members devote less time to research (self-imposed deadlines) than to teaching and service (externally imposed deadlines). In contrast, faculty (predominantly male) who have a partner to take caretaking responsibilities devote more time to research as opposed to teaching and service activities (Jacobs and Winslow, 2004; Misra et al., 2012). Indeed, married men tend to exhibit more research productivity (Bellas, 1992; Bellas and Toutkoushian, 1999) and occupy higher-level positions in academic institutions than unmarried men and both married and unmarried women (Bellas, 1992). Females are also more likely than males to be asked and allocate time to out-of-classroom activities, such as student advising of both academic and personal issues (El-Alayli et al., 2018), which are rarely taken into consideration for tenure and promotion decisions (Babcock et al., 2017).

Despite the wealth of information on gender disparities in time allocation, relatively few studies have included race as a factor when investigating time allocation to academic activities. Some of these studies have suggested that race is not a critical factor when looking at time allocation (e.g., Elmore and Blackburn, 1983; Russell et al., 1991). However, the usually small sample of individuals from racial and ethnic minorities underrepresented in academia (hereafter *underrepresented minorities* or *URMs*) may obscure differences between URM and nonURM individuals in their time allocation (see Bellas and Toutkoushian, 1999, for discussion). In a study analyzing the 1993 National Survey of Postsecondary Faculty (IPEDS; National Center for Education Statistics, 1994), Bellas and Toutkoushian (1999) observed that Black faculty spent less time on teaching activities and more time in service activities than White faculty. The critical variable appeared to be the likelihood of doing “paid” vs. “unpaid” work, with women and URMs receiving more requests and being more willing to engage in unpaid activities. Furthermore, women and URMs may not be part of the networks that increase publication success (Exum, 1983); indeed, Black and female faculty tend to produce fewer publications than White male faculty (Betsey, 2007).

Although service is usually the least important criterion in promotion and tenure decisions (e.g., Blackburn and Lawrence, 1995; Washburn-Moses, 2018), it can be difficult for faculty to balance the activities that “matter” for tenure and promotion (i.e., research and teaching) and the service responsibilities that are essential for shared governance (Baez, 2000). Women are most likely to be asked to complete and engage in service activities, especially those internal to the institution (Guarino and Borden, 2017; O’Meara et al., 2017a,b). The increasing interest in diversity, equity, and inclusion may have had the unfortunate effect that URMs receive excessive requests for service, including participation in committees and student advisement. The direct consequence of this higher investment in service is that time resources cannot be allocated to research and teaching activities (i.e., these activities are mutually exclusive; Fairweather, 1993; Dey et al., 1997). However, due to the requirement to fulfill teaching duties, increased time allocation to service is usually associated with reduced time allocated to research (Bellas and Toutkoushian, 1999; Betsey, 2007). Some theorists have correctly argued that service provides URMs with status that empowers them as agents of change in their institution and should be weighed as equally important as service and research (Baez, 2000). However, the reality is that tenure and promotion decisions at most US universities (but not necessarily at primarily teaching colleges) continue to be based on research productivity and teaching effectiveness, with the former weighing more heavily on tenure and promotion decisions (Easterly and Pemberton, 2008). Furthermore, faculty at most universities are pressured to actively seek external funding (Kleinfelder et al., 2003), as exemplified by faculty search ads, which is difficult for primary caretakers who must make decisions as to whether to pursue external funding or allocate time to family responsibilities (Herbert et al., 2014).

Note that most studies on faculty time allocation have been conducted by surveying faculty at large, Primarily White institutions (PWIs; e.g., Misra et al., 2012; Dahm et al., 2015; O’Meara et al., 2017a,b), using national survey data but focusing on R1 institutions (e.g., Link et al., 2008; Anderson and Slade, 2016), or have collapsed Colleges and Universities in terms of whether they are 2- or 4-year institutions or whether they are PWIs or HBUs (Bellas and Toutkoushian, 1999; Perna, 2001; Jacobs and Winslow, 2004; Betsey, 2007; Winslow, 2010; BrckaLorenz et al., 2018; French et al., 2020). The present study focused on the specific context of Historically Black Colleges and Universities (HBCUs) and, specifically, HBUs. HBCUs surged in the US in the early 19th century to provide educational opportunities to Black and African-descent individuals who were not welcome at existing educational institutions. Starting with the founding of the African Institute (now Cheyney University) in 1827, and until 1964, HBCUs were established to serve students from Black and African descent, later extending this role to first-generation and low-income students (Thurgood Marshall College Fund, n.d.). Minority-serving institutions founded after 1964 are known as Primarily Black Institutions (PBIs). PBIs are institutions characterized by, “at least 40% African-American students, minimum of 1,000 undergraduates, have at least 50% low-income or first-generation degree seeking undergraduate students, and have a low per full-time undergraduate student expenditure in comparison with other institutions offering similar instruction” (Thurgood Marshall College Fund, n.d.). HBCUs have historically been student-centered (Fountaine, 2012) and community-oriented (Gasman, 2013). The value of HBCUs has been frequently questioned (Wilcox et al., 2014), with growing pressure to serve non-Black students (e.g., Outcalt and Skewes-Cox, 2002). However, they are still relevant as producers of Black leaders (Albritton, 2012), are among the leading institutions producing Black engineers (Boyington and Moody, 2021), and more than 30% of all Black science and engineering doctorates (UNCF, n.d.). Importantly, HBCUs are not homogeneous, ranging from 2-year institutions to doctoral and professional degree-awarding institutions, from elite schools with competitive admissions to open-admission institutions, as well as diverse levels of funding, student profiles, ranking, and Afro-centric curricula. Thus, analyses of HBCUs that “lump” all institutions into a single category fail to account for the diversity in their institutional missions (Arroyo and Gasman, 2014; Wilcox et al., 2014).

The rising costs of education have led many institutions of higher education to experience financial challenges (Betsey, 2007; Gasman and Commodore, 2014). Even with the signing of a Presidential Executive Order increasing federal funding to HBCUs (2017), the Congress HBCU PARTNERS bill (2021), and recent private donations in excess of $800 million to minority-serving institutions including several HBCUs, the financial gap between HBCUs and PWIs is still large. HBCUs tend to have small endowments, receive less state funding than larger PWIs, and depend heavily on fundraising, and these funding woes cannot be compensated with tuition increases that are incompatible with serving minority, first-generation, and low-income students (Gasman, 2013; Gasman and Commodore, 2014). Financial pressures have led to a reduction in tenure-track faculty hiring or hiring freezes, an increase in adjunct faculty, and reliance on online programs. Furthermore, financial struggles directly and indirectly affect faculty’s time allocation, as the institutions try to maintain their educational and community service missions. HBCU faculty teach an average of four courses per semester, receive salaries that are significantly lower than peers at other institutions, and are expected to mentor and assist students, especially those from disadvantaged backgrounds (Gasman, 2013). These requests may decrease time otherwise allocated to research, considering that the number of research products from HBCU faculty is lower on average than from faculty at PWIs, although overall career productivity is similar to faculty at PWIs (Betsey, 2007). Contrary to Link et al.’s observation of decreased research productivity as faculty advance in rank, Betsey (2007) observed that HBU faculty productivity increases as they advance in rank, probably due to increased teaching experience or reduced teaching loads. However, these observations do not take into consideration faculty attrition, given that individuals who fail to exhibit research productivity may not attain tenure and progress in the academic ranks.

The present study surveyed STEM faculty at five HBUs in the southeastern United States, asking them to estimate their current time allocation, their ideal time allocation, and their expected time allocation. The goal was to determine not only how their time is used, but also their perceived constraints to allocate their time in a way that is convenient for the progress of their careers, and whether they were well informed about the time distribution that was expected from their institutions. We will discuss faculty estimations of time allocations in light of the tenure and promotion guidelines published in the faculty handbooks at the HBUs that participated in this study. Each institution defined productivity in the areas of teaching, research, and service/outreach in accordance with their institutional values. Although all institutions describe research expectations (peer- and non-peer-reviewed publications and peer evaluations), only the larger institutions specifically mention grant proposals and attainment of external funding as essential to demonstrate research productivity. The smaller institutions appear to emphasize teaching excellence, including student advisement, curriculum/course development, and mentorship. Service to the University is also emphasized, with some institutions encouraging “unpaid” service activities, such as attending informal events on campus (e.g., athletic events).

Previous studies of faculty time allocation have analyzed faculty across disciplines, specifically the humanities and STEM, but despite observing some differences across fields, data have been interpreted in terms of another variable (e.g., gender, Winslow, 2010). Some studies highlighting research productivity have collapsed data from faculty across the arts and sciences, consistent with the organization of Colleges at many institutions (e.g., Bellas and Toutkoushian, 1999; French et al., 2020), or focused on STEM faculty (e.g., Misra et al., 2012; Anderson and Slade, 2016). Our study focused specifically on faculty in four STEM fields (biology, engineering, mathematics, and agricultural sciences), which represent different aspects of STEM, and which have expectations of research productivity for successful attainment of tenure and/or promotion. STEM faculty at HBUs are of particular interest because URMs make up approx. 30% of the US population but only about 9% of STEM faculty in the US (National Center for Science and Engineering Statistics, 2017) and a large proportion of these URM STEM faculty are housed at HBUs (Strauss, 2015; Gasman, 2021). Thus, as a whole HBUs have a more diverse professoriate than other institutions of higher education (Strothers, 2014) and are a unique environment in which URMs are *not* a minority. Because of these unique characteristics, it is possible that some of the constraints known to determine faculty effort distribution do not apply to faculty at HBUs. For example, it is possible that URM faculty do not experience the pressures related to tokenism they experience at PWIs or that untenured faculty allocate their effort in a manner consistent with the teaching mission of HBUs. However, the experiences of STEM faculty at HBUs have not been the focus of research on faculty time allocation, resulting in a void in our understanding of the reasons that foster or impede the success of HBU STEM faculty.

Time allocation has been viewed as a determinant of job satisfaction. In lieu of asking participants directly how satisfied they are with their job, we chose to ask them to estimate their *ideal* time allocation. This was intended to provide a measure of the deviation between what faculty expect their job to be and their actual work responsibilities, an indirect measure of professional satisfaction. External pressures are known to decrease teaching effectiveness (BrckaLorenz et al., 2018), change faculty behavior and reduce job satisfaction (e.g., Anderson and Slade, 2016), and increase turnover intentions (French et al., 2020). Finally, estimates of *expected* time allocation, faculty’s view of what their institution expects them to do, provide an idea of what the faculty perceive they ought to do to meet the requirements of their position. Taken together, these measures can provide a rough picture of how well institutional expectations match the “ideal job” for HBU faculty, and the extent to which external pressures forces faculty to deviate from that ideal.

## Materials and Methods

### Participants and Procedure

Participants were STEM faculty at five HBUs in the southeastern United States. They were invited to participate in the study *via* an email solicitation sent to all STEM faculty having a tenured or tenure-track position at their institutions. A total of 473 individuals were invited to participate in the study. Survey return rate was 18% (*n*=84). Individuals electing to participate were provided with an informed consent, and only participants agreeing to the terms of this informed consent progressed to the study. Participation required completing an online survey, which included demographic questions, as well as questions about their effort distribution. Participation was incentivized *via* monetary compensation in the form of gift cards. All procedures described below were carried out with approval of the Oakland University Institutional Review Board (IRB) and were conducted in accordance with the guidelines of the 1964 Helsinki Declaration and its later amendments.

The participating institutions were all doctoral-granting institutions, with three of them labeled as “Doctoral Universities, higher research activity,” and two of them labeled as “Masters colleges and universities, larger programs” according to the Carnegie Classification of Institutions of Higher Education (2018). All of the participating institutions offer post-baccalaureate degrees in four selected fields, which represent the four “hard” science areas of the Biglan (1973) model: hard-life-pure (biology/microbiology), hard-life-applied (agronomy/agricultural sciences/agricultural economics), hard-nonlife-pure (mathematics/statistics), and hard-nonlife-applied (engineering/information sciences). Currently, 107 institutions are designated as HBCUs (three of these institutions were closed at the time this study was conducted), and 14 of those institutions offer all four of the disciplines selected for study; thus, the five participating institutions represent 5% of all HBCUs and 36% of HBUs offering all four of the selected disciplines. The participating HBUs were all located in neighboring states, providing similar social contexts for the institutions. The study consisted of a survey, which was administered in alternate semesters (Round 1, *n*=48; Round 2, *n*=46). In order to protect the anonymity of responses, participants were asked to create a survey ID, which was used to identify individuals who had participated in Round 1 in order to avoid duplication of data in Round 2. Ten individuals completed the survey in both Rounds 1 and 2, and for all questions that were repeated across surveys, only their most recent response was used for analyses. Thus, all data analyses reflect one response from each participating faculty member.

### Measures

#### Current and Ideal Time Allocation

As part of a larger research project investigating other aspects of faculty experiences at HBUs, participants were asked to estimate the percentage of their time that is allocated to each of the required professorial activities: research, teaching, service, and outreach. The question was a “zero-sum” question, so that participants had to estimate all of their work time in a week (100%) how much (in percentage) was allocated to each activity; the sum of all time allocation had to add up to (but could not exceed) 100% (the total amount of time worked in a week). The prompt was as:

*Estimate the number of hours per week that you devote to each of the following activities. Please read the descriptors carefully and select the answer that is most consistent with your actual experience in an average week*.

***Research:** time devoted to research, literature reviews, laboratory time, writing papers, writing grants, and completing administrative duties directly related to research*.

***Teaching:** time devoted to class preparation, classroom or online teaching, grading, office hours and advising, and administrative duties related to teaching*.

***Service:** time devoted to serving in committees or functions that serve your department or academic unit, college or school, university, professional organizations, and your profession in general*.

***Outreach:** time devoted to expanding the impact of your field and institution to benefit the community at large. If your institution categorizes outreach as a form of research, teaching, or service for the purposes of promotion and tenure and/or faculty evaluation, please include the time you devote to these activities under the category that is consistent with your institutional policies*.

*What is your current time distribution, as represented by a proportion or percentage? Note that you will need to allocate time in such a way that it adds up to 100% across all categories. If one of the categories does not apply to your appointment, leave it as a zero (0)*.

Note that asking about time allocation using a zero-sum format normalizes potential wide differences in the estimation of the number of hours worked in a week. Following the estimation of time allocated to each activity in a working week, participants were asked to provide an estimation of the percentage of time they would like to allocate to each activity (their “ideal” time distribution). The prompt for this estimation was as:

*What would be your ideal time distribution, as represented by a proportion or percentage? Note that you will need to allocate time in such a way that it adds up to 100% across all categories. If one of the categories does not apply to your appointment, leave it as a zero (0)*.

To better understand the pressures imposed on time allocation, participants were asked whether obtaining external funding to cover their research expenses was required by their academic unit in order to successfully attain tenure and promotion. Current and ideal time allocation questions, as well as the research requirement question, were included in Surveys 1 and 2.

#### Time Allocation in Preparation for Tenure and Promotion

A sub-sample of participants was asked what percentage of their time should be allocated to each activity in accordance to their institutional tenure and promotion policies. The question was added during the second round of surveying in order to better interpret the data obtained for the estimates of current and ideal time allocation. The sample participating in both rounds of surveys was roughly equivalent (50 and 47.8% untenured, 39.5 and 37.2% female, and 44.7 and 28.3% URM faculty for Rounds 1 and 2, respectively). They were asked to estimate the time those policies required that they allocate to each of their academic activities. The prompt was as:

*Based on your department/academic unit's current promotion and tenure guidelines, what should be the time distribution of a faculty member in your department? Note that you will need to allocate time in such a way that it adds up to 100% across all categories. If one of the categories does not apply to your appointment, leave it as a zero (0)*.

Untenured faculty were further asked whether their current time distribution was adequate to obtain tenure and promotion, which they rated in a 4-point scale (definitely not, probably not, probably yes, and definitely yes). Further, all faculty were asked whether their academic unit’s actual criteria for tenure and promotion were consistent with those specified in the faculty handbook, an informal convention that applied to their academic unit, or a guess they had because they were not sure about the actual requirements.

### Statistical Analyses

Current and ideal time allocation data were analyzed using ANOVAs. Estimate (current vs. ideal) and activity (research vs. teaching vs. service vs. outreach) were entered into the ANOVA as within-subjects factors, whereas demographic variables (tenure status, gender, and representation in STEM) were entered into the ANOVA as between-subjects factors. The research questions led to expected interactions, and whenever an interaction was observed, the source of the interaction was assessed using univariate tests of significance. The large number of univariate tests that resulted from each analysis can increase the likelihood of a Type I error. This issue was addressed using a False Discovery Rates correction, in which the value of *p* for each comparison was adjusted using the Benjamini and Hochberg (1995) method. Briefly, this correction estimates that, if the level of significance is set at *α*=0.05, there is a 5% likelihood that a comparison reveals a “false positive” (Type I error). The method ranks the values of *p* for all comparisons based on their value and corrects the significance level (*p*_adj_) using the formula:


$$p_{adj}=\left\{\frac{totalnumberof\;p\;values}{p\;value\;rank}\right\}$$

Comparisons between expected and current time allocation used Welch’s *t*-test. Welch’s *t*-test (rather than student’s *t*-test) was used for analyses due to the difference in size and variance of the samples compared (Ruxton, 2006). Welch’s *t*-test adjusts degrees of freedom by dividing each group’s variability by the group’s size (rather than using a pooled variability score).

Comparisons among frequencies were conducted using the chi-square (*χ*^2^) statistic.

## Results

Descriptive statistics for demographic information are presented in Table 1. Data are not divided by institution or academic unit in order to ensure the anonymity of the participants’ responses. Five participants did not provide information about their gender and were excluded from the gender analyses.

**Table 1:** Demographic information of the participant sample.

Gender	Representation	Tenured	Untenured	Total
Male	URM^*^	7	6	49
	nonURM^**^	17	19	
Female	URM	8	7	30
	nonURM	7	8	
Declined to provide gender	URM	2	0	5
	nonURM	2	1	
	Total	43	41	84

* *URM: n=30*;

** *nonURM: n=54*.

### Current and Ideal Time Allocation

**Figure 1:**
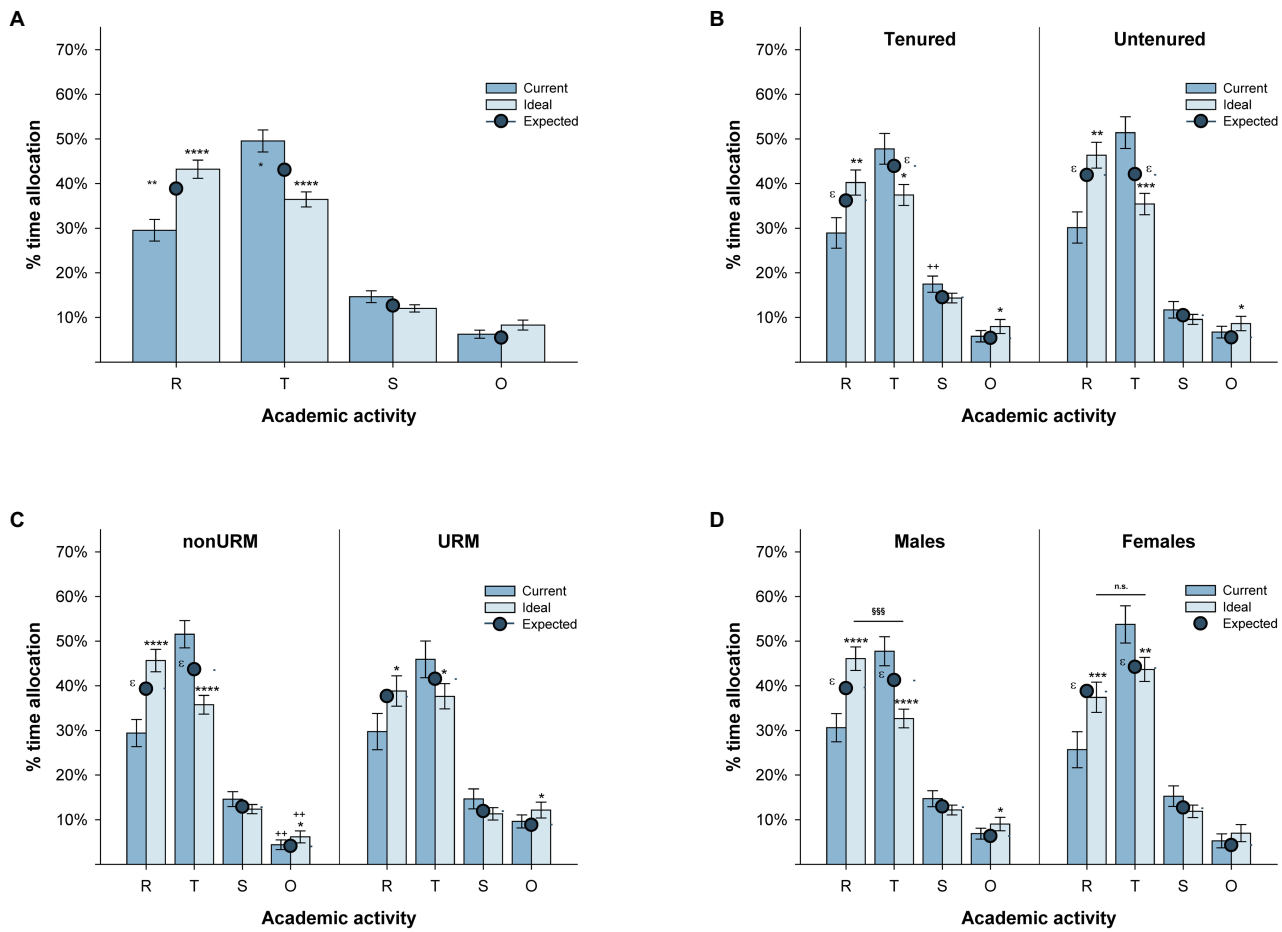
Percent time allocation. Time allocation for all faculty (**A**), faculty’s tenure status (**B**), representation (**C**), and gender (**D**). In the horizontal axis, R, research; T, teaching; S, service; and O, outreach. For comparisons between current and ideal time allocation, ^*^p_adj_<0.05, ^**^p_adj_<0.01, ^***^p_adj_<0.005, and ^****^p_adj_<0.001. For comparison between ideal time allocation to research and teaching, ^§§§^*p*_adj_<0.001 and n.s, not significant. For comparisons between current and expected time allocation, ^ɛ^*p*<0.05. Brackets represent standard error of the mean. See text for further details.

Time allocation responses were obtained from 84 participants. A 2(estimate: current vs. ideal)×4(activity: research vs. teaching vs. service vs. outreach) ANOVA revealed a main effect of activity, *F*(3, 249)=95.09, *p*<0.001, and an Estimate × Activity interaction, *F*(3, 249)=42.31, *p*<0.001 (Figure 1A). The interaction was further analyzed with univariate tests, which revealed that participants rated the time currently allocated to teaching and service to be higher than they would like to allocate to those activities, *p*_adj_<0.001 and 0.05, respectively, and the time currently allocated to research and outreach to be lesser that they would like to allocate to those activities, both *p*_adj_<0.001.

#### Tenured vs. Untenured Faculty

A 2(estimate)×4(activity)×2(tenure status: tenured vs. untenured) ANOVA revealed a main effect of activity and an Estimate × Activity interaction, *F*s(3, 246)=95.43 and 49.50, *p*s<0.001, respectively (Figure 1B). The main effect of tenure status and all interactions with this factor were not significant, all *p*s>0.17. However, the ideal service allocation was higher for tenured than untenured faculty, *p*_adj_<0.01. When other activities were considered, both tenured and untenured faculty rated their current time allocation to research as lower than their ideal time allocation for research (both *p*_adj_<0.01), their current time allocation to teaching as higher than their ideal time allocation to teaching (*p*_adj_<0.05 and 0.005 for tenured and untenured faculty, respectively), and their current time allocation to outreach as lower than their ideal time allocation to outreach (both *p*_adj_<0.05). Thus, tenured and untenured faculty estimate their current time allocation to research and teaching similarly, and their ideal time allocation would increase research time and decrease teaching time in a similar manner. However, tenured faculty seem willing to allocate more of their time to service than untenured faculty.

#### NonURM vs. URM Faculty

A 2(estimate)×4(activity)×2(representation: URM vs. nonURM) ANOVA revealed a main effect of activity and an Estimate × Activity interaction, *F*s(3, 246)=82.99 and 34.11, *p*s<0.001, respectively (Figure 1C). However, unlike the tenure analysis, there was a three-way, Estimate × Activity × Representation interaction, *F*(3, 246)=2.95, *p*<0.05. URMs estimate their current time allocating to outreach to be higher than nonURMs, and their ideal time allocation also includes more outreach than nonURMs, both *p*_adj_<0.05. Comparisons between current and ideal time allocation revealed that both URMs and nonURMs would desire to devote more time to research (*p*_adj_<0.05 and 0.001, respectively) less time to teaching (*p*_adj_<0.05 and 0.001, respectively), and more time to outreach (both *p*_adj_<0.05). Thus, although nonURMs and URMs have similar patterns in their current and ideal time allocations, URMs seem to be committing more time to outreach than nonURMs.

#### Male vs. Female Faculty

Five participants declined to provide their gender; thus, the gender analyses are based on 79 participants. A 2(estimate)×4(activity)×2(gender: male vs. female) ANOVA revealed a main effect of activity, *F*(3, 231), *p*<0.001, an Estimate × Activity interaction, *F*(3, 231), *p*<0.001, and an Activity × Gender interaction, *F*(3, 231), *F*(3, 231)=2.85, *p*<0.05 (Figure 1D). There were no gender differences in current time allocation (all *p*_adj_>0.37). However, when ideal time allocation was compared, males wished to devote less time than females to teaching, *p*_adj_<0.005. Both males and females would like to allocate more time than they currently do to research (*p*_adj_<0.001 and 0.005, respectively), and less time to teaching (*p*_adj_<0.001 and 0.01, respectively). Males would also like to allocate more time than they currently do to outreach (*p*_adj_<0.05). Importantly, when the current time allocation to research and teaching was compared, both males and females reported allocating more time to teaching than research (*p*_adj_<0.05 and 0.005, respectively), whereas the ideal time allocation to teaching vs. research differed for males but not for females (*p*_adj_<0.01 and >0.35, respectively). Thus, although males and females allocate about the same amount of time to their professorial activities and would like to allocate more time to research and less time to teaching than they currently do, males’ ideal distribution of time includes allocating more time to research than teaching, whereas females’ ideal distribution of time includes devoting equivalent time to teaching and research, and significantly more time to teaching than males.

### Time Allocation and the Path to Tenure

#### Expected Time Allocation

A sub-sample of participants (*n*=41) was asked to estimate their academic unit’s expectations of time allocation in order to grant faculty tenure and promotion. There were no interactions between expected time allocation and tenure status, representation, or gender (all *p*s>0.18), suggesting that faculty across all demographics have a consistent view of their institution’s expectations for research, teaching, service, and outreach. Thus, data were collapsed across these factors. A one-way ANOVA revealed that expected time allocation to academic responsibilities was rated differently, *F*(3, 132)=70.90, *p*<0.001. Expected time allocations to teaching and research were higher than the expected time allocations to service and outreach, and expected time allocation to service was higher than to outreach, all *p*_adj_<0.001. However, expected time allocation to teaching and research did not differ, *p*_adj_>0.37. Thus, faculty estimated that they should allocate similar amounts of time to their teaching and research, and less time to their service and outreach, although outreach was viewed as the less valued activity.

Note that both tenured and untenured faculty were asked to answer this question and their responses entered into the analyses because this allowed for a rough determination of whether faculty on the tenure track (the faculty who are trying to meet expectations) had similar views of the institution’s expectations as faculty who had already gone through tenure and promotion (the faculty who evaluate those expectations). The lack of differences in estimation of expected allocation between tenure-track and tenured faculty suggests a consistent view of institutional expectations to be met by faculty who successfully attain tenure and promotion.

#### Alignment of Current and Ideal Time Allocation to Expected Time Allocation

Current time allocations were compared to the expected time allocation using one-tailed Welch’s *t*-tests (see Figures 1A–D). For the overall sample, time allocated to research was estimated to be lower than expected, *t*(108)=2.60, *p*<0.01, and time allocated to teaching was estimated to be higher than expected, *t*(120)=1.94, *p*<0.05. Tenured and untenured faculty estimated that they allocate less time to research than expected, *t*(63)=2.00 and *t*(46)=1.84, both *p*s<0.05. However, only untenured faculty estimated the time allocated to teaching to be higher than expected, *t*(56)=1.69, *p*<0.05. NonURM faculty estimated that their time allocation to research was lower, *t*(71)=2.18, and their time allocation to teaching was higher, *t*(81)=1.91, than expected (both *p*s<0.05). Surprisingly, URM faculty did not exhibit any discrepancies between their current time allocation and the time allocation expected by their institution, all *p*s>0.08. Finally, both males and females estimated that their time allocation to research was lower than expected, *t*(58)=1.77 and *t*(39)=2.26, respectively, both *p*s<0.05. However, only females estimated their time allocation to teaching to be higher than expected, *t*(41)=1.87, *p*<0.05. These results suggest that, although faculty overall consider that they should be allocating more time to research and less time to teaching, URMs consider that they are currently allocating the time that is expected to both of these activities. Across all levels of representation (URM and nonURM) females (but not males) and untenured faculty appear to estimate that the time allocated to their teaching (which in our survey included student advising and supervision) is not consistent with what they should be allocating to their professorial activities in order to attain tenure.

When ideal and expected time allocations were compared, the overall sample estimated their ideal time allocation to teaching to be lower than expected, *t*(90)=2.34, *p*<0.05, which was also the case for tenured, *t*(45)=1.86, and male faculty, *t*(46)=2.34, *p*s<0.05. The overall sample estimated their ideal time allocation to outreach to be higher than expected, *t*(123)=1.93, *p*<0.05, which was also the case for the nonURM faculty, *t*(77)=1.73, *p*<0.05. Notably, the demographics who viewed the time they currently allocate to teaching to be higher than expected (female and untenured faculty) would ideally adjust to those expectations, whereas the demographics who viewed their current allocation to teaching to be consistent with expectations (male and tenured faculty) would ideally allocate less time to teaching.

#### Information About Expected Time Allocation

The same sub-sample was asked whether the effort distribution that their academic unit expects from individuals seeking tenure and promotion is specified in the faculty handbook, based on informal expectations in their academic unit, or their best guess. Participants in the surveyed sample (*n*=45) were less likely to state that the handbook was the source of information for their estimates than informal expectations (*χ*^2^=6.02, *p*<0.05) and their best guess (*χ*^2^=14.70, *p*<0.001). Informal expectations did not differ as a source of information from their best guess (*χ*^2^=2.22, *p*>0.13). All comparison groups (tenure, representation, and gender) followed this same pattern. It is noteworthy that tenured faculty (who have a vote on tenure and promotion decisions) were also more likely to state that tenure and promotion decisions are more likely to be guided by evaluations of effort distribution based on informal expectations and their best guess than the faculty handbook (*χ*^2^s=7.11 and 10.10, *p*s<0.01).

#### Expectation of Research Productivity

Participants were asked whether obtaining external funding was required to successfully attain tenure and promotion. Two participants did not answer this question; thus, the data below are based on 82 responses. Eighty nine percent of the surveyed faculty considered that obtaining external funding was required to obtain tenure and promotion. There were no differences in expectation of funding as a requirement for tenure and promotion among tenured and untenured faculty (*χ*^2^=0.06, *p*>0.83), nonURM and URM faculty (*χ*^2^=0.25, *p*>0.62), or male and female faculty (*χ*^2^=1.49, *p*>0.22).

#### Reasons for Current Time Allocation

**Figure 2:**
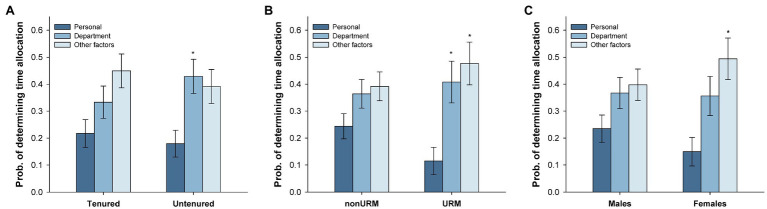
Perceived reasons for current time allocation. Tenured vs. untenured (**A**), nonURM vs. URM (**B**), and male vs. female (**C**) faculty estimated the extent to which their current effort distribution was guided by personal preferences, departmental pressures, or other institutional factors (e.g., institutional policies and available resources). ^*^p_adj_<0.05 for comparisons between personal preferences, and departmental and institutional pressures. Brackets represent the standard error of the mean.

If obtaining external funding is required to obtain tenure and promotion, and faculty would like to devote more time to research than teaching, one may wonder why they allocate their time the way they do. Participants (*n*=82) were asked whether their time allocation reflected their personal preference, the requirements of their academic unit, or other factors (e.g., institutional policies and availability of resources). Participants could select between one and three factors. Thus, their responses were weighed by the number of factors selected (e.g., if an individual selected all three factors, each factor received a weight of 0.33, whereas if they selected two factors, each factor received a weight of 0.5). A series of one-way ANOVAS were conducted to determine the impact of each of these motives for faculty time allocation. These ANOVAS revealed no differences among motives for effort distribution in tenured, nonURM, and male faculty, all *p*s>0.07. In untenured faculty, *F*(2, 78)=3.46, *p*<0.05, the probability of personal preference was lower than the probability of department/academic unit pressures, Tukey’s *p*<0.05 (Figure 2A). In URM faculty, *F*(2, 56)=4.99, *p*<0.05, the probability of selecting personal preference was lower than the probability of selecting department/academic unit and other factors as determinants of their time allocation, Tukey’s *p*s<0.05 (Figure 2B). Finally, in females, *F*(2, 56)=4.28, *p*<0.05, personal preference was rated lower than other factors, Tukey’s *p*<0.05 (Figure 2C).

## Discussion

The present study asked faculty at HBUs to estimate the percentage of their work time that was allocated to each of their academic responsibilities: research, teaching, service, and outreach. There is a wealth of studies investigating how faculty distribute their time (e.g., Elmore and Blackburn, 1983; Russell et al., 1991; Bellas and Toutkoushian, 1999; Jacobs and Winslow, 2004; Betsey, 2007; Link et al., 2008; Winslow, 2010; Misra et al., 2012; Dahm et al., 2015; Anderson and Slade, 2016; O’Meara et al., 2017a,b; El-Alayli et al., 2018; French et al., 2020). However, these studies have rarely been performed by directly surveying HBU faculty; rather, studies providing information on time allocation at HBUs have typically used national data, such as the IPEDs (e.g., Bellas and Toutkoushian, 1999; Perna, 2001; Betsey, 2007). The present survey asked HBU faculty to estimate the percentage of their work week that is allocated to each of four academic responsibilities (these percentages added to 100% of their work time), the percentage of their work time that they would ideally allocate to each academic responsibility if they had no external pressures, and the percentage of effort toward each academic responsibility their academic unit/institution expected from faculty in order to obtain tenure and promotion. We observed that, although faculty were consistent in their perspective of the effort distribution that was expected from them, the way in which they *do* (current) and *would like to* (ideal) allocate their time is not necessarily consistent with these expectations. It is noteworthy that all faculty (regardless of tenure status, representation, or gender), considered the time they currently devote to research to be lower than the time they would like to allocate to this activity, and the time they allocate to teaching to be higher than the time they would like to allocate to this activity. Untenured faculty and female faculty (who reported the largest discrepancies between the time they currently devote to teaching and research activities) view their current time allocation to research and teaching activities to deviate from what is required to obtain tenure and promotion, whereas tenured and male faculty only reported such deviation in the time allocated to research. Surprisingly, URM faculty did not view their current time allocation to any of their academic responsibilities as diverging from the time allocation expected by their academic unit/institution (see below for discussion). Consistent with HBUs’ tradition of extending education and research to the surrounding community, all faculty’s ideal allocation includes an increase from their current levels of outreach.

Historically Black Universities house a large proportion of the STEM URM professoriate in the US; however, little research has been devoted to understanding how faculty allocate their effort at these institutions. Despite HBU’s tradition of student service, the long-term research productivity of HBU faculty seems to mirror that of faculty at PWIs, suggesting that HBU faculty allocate their effort in a way consistent with the expectations of large research institutions. BrckaLorenz et al. (2018) concluded that faculty fit one of five profiles based on how they allocate their time to research, teaching, and service: research-heavy (high research, moderate teaching, and low service), teaching-heavy (low research, high teaching, and low service), service-heavy (low research, moderate teaching, and high service), classic (moderate research, high teaching, and low service), or moderate load [low research, moderate teaching, and low service; a sixth, dual teaching-service profile was later suggested by French et al. (2020)]. In their sample, the number of hours of work reported by faculty varied between 25 and 53.5h/week with the lowest number of hours reported by moderate load, followed by teaching-heavy, research-heavy, service-heavy, and classic faculty (in increasing order). The aggregated profile for our HBU faculty’s current time allocation (Figure 1A) is most consistent with a teaching-heavy profile; however, in the sampled faculty’s estimation, the profile expected by their institution is a classic profile (equivalent allocation of effort to teaching and research). Note that a difference between BrckaLorenz et al. and French et al.’s studies and the present study is that our participants were not asked to estimate a number of hours invested in each academic activity, but rather the proportion of time they allocated to each activity. However, the fact that a profile consistent with their defined profiles emerged suggests that the measures may be comparable.

One could view the similarities in current time allocation across faculty demographics as an equivalent effort to fit the profile expected by their institution and the observed differences as the result of uneven pressures on some of these demographics. Even though URM faculty are not a minority at HBUs, they may experience more pressures to mentor URM students than nonURM faculty, a pressure that may be increased in times of financial uncertainty (Gasman, 2013; Gasman and Commodore, 2014). Financial uncertainty may also lead to increased burdens on untenured faculty who may resort to taking extra service commitments to increase their profile at their institution. Race and gender are known to be associated to time allocated to out-of-classroom activities (Bellas and Toutkoushian, 1999), and the difficulties experienced by female faculty of color at PWIs are also experienced at HBUs (Blackshear and Hollis, 2021). Although the present study cannot yield conclusions regarding the reasons why race, gender, and their intersection yield differential time allocation profiles, we assume that the determinants of such time allocation in HBU and PWI faculty are mediated by the intersection of personal variables and the institutional context.

Another interesting observation derived from this study is that faculty did not view their institution’s faculty handbook as the source of expectations for tenure and promotion decisions. Rather, they stated that tenure and promotion decisions were based mostly on informal expectations or their best guess of what those expectations were. This suggests that, without continuous feedback from their academic unit, faculty on the tenure-track may be distributing their time in a way that is inconsistent with established policies or, if adjusting their time allocation to established policies, their effort may not be consistent with the informal expectations of faculty productivity. The observation that tenured faculty (who make tenure and promotion decisions) also reported a lack of reliance on the faculty handbook suggests that there may not be clear guidelines for faculty seeking tenure. One reason for this lack of confidence in the faculty handbook may be related to the rotation of administrative personnel that is commonly observed in HBUs (e.g., Gasman, 2013). Another reason may be informal practices that have become established practices. For example, the faculty handbooks that we reviewed only mentioned submitting proposals or attaining external funding as expected from individuals seeking tenure at the larger institutions; nonetheless, faculty at all institutions reported that obtaining external funding was required to successfully obtain tenure and promotion.

Historically Black Colleges and Universities continue to be the institutions serving the students in greatest need of support and advisement, even in the face of the financial and administrative challenges with which they must contend (e.g., Gasman, 2013). However, their faculty are often evaluated (or *perceive* that they are evaluated) using a model developed for research-intensive institutions (*cf*. Jacobson, 1992; also see Fairweather, 2002). In this model, research is the most critical determinant of tenure and promotion decisions, whereas other activities, such as service, are viewed as less important (Baez, 2000). Not surprisingly, faculty in this study consistently viewed the time they would ideally allocate to research as significantly higher than what they currently allocate. Some demographics in the surveyed faculty also reported constraints in their time allocation, with untenured faculty, women, and URMs viewing factors other than their personal preferences as determining the way in which their time was allocated. Notably, the authors could not find any references to time allocation to outreach (to which the surveyed faculty would like to devote more time). The desire of HBU faculty to engage in outreach suggests that HBUs continue their historical function of service to the community and attract community-oriented individuals into its professoriate (Blake, 2018). This community service academic responsibility seems to be largely ignored in studies of faculty at PWIs, possibly grouping outreach activities into the research or service categories.

Some of our findings are consistent with previous literature on faculty time allocation. For example, females were more likely to view teaching as a rewarding part of their appointment, as reflected in their desired allocation of equivalent amounts of time to teaching and research (Bellas and Toutkoushian, 1999; Link et al., 2008; Winslow, 2010; Misra et al., 2012; Dahm et al., 2015; French et al., 2020). URMs in our sample reported equivalent proportion of time allocated to service activities as nonURM faculty, which is inconsistent with previous reports (e.g., Bellas and Toutkoushian, 1999). A possibility is that studies conducted at PWIs (or when a large number of institutions analyzed are PWIs) reflect “tokenism,” or the fact that URMs are more likely to receive requests to participate in service activities. This tokenism may be less prevalent in HBUs, in which there is a larger representation of URM faculty (e.g., Gasman, 2013). Notably, URM faculty were the only demographic that reported no deviations from their current time allocation and the time allocation expected by their institution. This may reflect URMs’ greater satisfaction with their role at the HBU than may be experienced by nonURM faculty. Indeed, Black faculty tend to report a better “fit” to professorial roles at HBUs than at PWIs (e.g., Mangan, 2015).

In summary, the present study should be considered a pilot investigation into the idiosyncrasies of faculty time allocation at HBUs. Considering the cultural and historical context of HBUs, the pressures for effort distribution imposed by the needs and function of the institution may lead to a better understanding of satisfaction, recruitment, and retention of faculty at HBUs.

## Limitations and Pathways To Further Research

The largest limitation of the present study is that the sample size was large enough to analyze differences among faculty based on some demographic characteristics, but not large enough to conduct intersectional analyses. For example, URM female faculty may have challenges that are not shared by URM male faculty, and URM male faculty may encounter challenges that are not shared by nonURM male faculty (for a recent study investigating the unique challenges faced by Black women pursuing science and technology degrees, see Nguyen et al., 2021). Our sample was greatly skewed toward nonURM faculty in the tenure track (i.e., untenured), which may reflect a selection bias, since we could only analyze the data from those individuals who returned the surveys. However, this oversampling of nonURM faculty may reflect a gradual change in the composition of the professoriate at HBUs. For example, over the past 4years, three of the participating institutions (which serve a total of approx. 22,000 students) collectively had less than 18 Black faculty in the tenure track, a number of which were not US Nationals. This is significant, as it may reflect HBUs’ competition with PWIs for Black faculty, a growing problem over the last 20years (e.g., Jackson, 2002; Mangan, 2015). Asking faculty to estimate how they allocate their time is also fraught with uncertainty and biases, and a more accurate approach would involve journaling of time investment in each academic responsibility (e.g., O’Meara et al., 2017b). Thus, the present data may reflect a subjective perception of investment, which may be exaggerated for laborious activities and minimized for preferred activities. Finally, a study including perceptions of leadership on faculty time allocation and tenure and promotion policies would allow for a better definition of institutional expectations of their faculty effort distribution.

## Conclusion

Most research on faculty time allocation has been conducted in institutions other than HBUs. The present study revealed that, consistent with previous research on faculty time allocation, STEM HBU faculty allocate more time than they desire to teaching and less time than they desire to research. The way in which these faculties currently allocate their time and how they wish to allocate their time varies depending on certain demographic factors (based on tenure status, gender, and representation), despite the fact that all faculty groups analyzed have consistent views of what time allocation is expected by their institution. Current time allocation appears to be largely determined by external pressures rather than personal preferences, and the surveyed faculty reported a lack of clarity on the criteria used to make tenure and promotion decisions at their institution.

Although limited by its relatively small sample size, this study provides a preliminary view of STEM faculty time allocation at HBUs and highlights the fact that, although faculty can adjust to the context of their institution, there are still steps that could be taken to increase their success at the HBU. Furthermore, an understanding of how faculty allocate their effort as opposed to their preferred effort allocation could be used as the basis for designing policies aimed at recruiting and retaining quality STEM faculty at HBUs. The surveyed faculty fit the “teaching-heavy” profile for time allocation, but their ideal profile is consistent with the “classic” profile expected by their institutions. “Teaching-heavy” faculty tend to report the lowest levels of job satisfaction and the highest turnover intentions, whereas “classic” faculty report high levels of job satisfaction and low turnover intentions (French et al., 2020). Thus, increasing the clarity of tenure and promotion guidelines and ensuring that reviewing bodies adhere to those guidelines could be a first step that facilitates faculty’s adjustment to the profile expected by the institution, as well as increase job satisfaction and faculty retention.

## Data Availability Statement

The raw data supporting the conclusions of this article will be made available by the authors, without undue reservation.

## Ethics Statement

The studies involving human participants were reviewed and approved by the Institutional Review Board (IRB), Oakland University. The patients/participants provided their written informed consent to participate in this study.

## Author Contributions

ME and MQ are the PIs of the grants funding the research. ME contributed to the design of the study, reviewed the literature relevant to the development of the measures used in this study, wrote the initial draft of all sections of the manuscript, integrated feedback from co-authors through the editing process, and was responsible for the data analyses and discussion of the study. ZB assisted with literature review and data collection, and provided feedback on the manuscript. MQ contributed to the design of the study, reviewed and provided feedback on the manuscript, and participated in developing the discussion. CK assisted with literature review and data collection, and reviewed and provided feedback on the manuscript. FA assisted with statistical analyses and provided feedback on the manuscript. All authors contributed to the article and approved the submitted version.

## Funding

This material is based upon work supported by the National Science Foundation Awards #1820961 and #1820981. Any opinions, findings, and conclusions or recommendations expressed here are those of the authors and do not necessarily reflect the views of the National Science Foundation.

## Conflict of Interest

The authors declare that the research was conducted in the absence of any commercial or financial relationships that could be construed as a potential conflict of interest.

## Publisher’s Note

All claims expressed in this article are solely those of the authors and do not necessarily represent those of their affiliated organizations, or those of the publisher, the editors and the reviewers. Any product that may be evaluated in this article, or claim that may be made by its manufacturer, is not guaranteed or endorsed by the publisher.
